# Full-space spin-decoupled versatile wavefront manipulations using non-interleaved metasurface

**DOI:** 10.1515/nanoph-2023-0171

**Published:** 2023-06-19

**Authors:** Chaohui Wang, He-Xiu Xu, Guangwei Hu, Yi Liu, Tong Liu, Kun Wang, Fan Zhang, Shuo Xu, Jian Xu, Zhichao Pang

**Affiliations:** Air and Missile Defense College, Air Force Engineering University, Xi’an 710051, China; School of Electrical & Electronic Engineering, Nanyang Technological University, 50 Nanyang Avenue, Singapore 639798, Singapore

**Keywords:** full-space, multifunctional metasurface, spin-decoupled, transmission, wavefront manipulations

## Abstract

Achieving multifunctional wavefront manipulations of waves with a flat and thin plate is pivotal for high-capacity communications, which however is also challenging. A multi-layer metasurface with suppressed mode crosstalk provides an efficient recipe primarily for circular polarization, but all multiple functionalities still are confined to locked spin states and modes. Here, a multifunctional metasurface with spin-decoupled full-space wavefront control is reported by multiplexing both linear momentum and frequency degree of freedom. We employed vertically cascaded quadrangular patches and crossbars to integrate both geometric and dynamic phases and realized four channels between two spin states and two frequencies in distinct scattering modes (transmission and reflection). For verification, a proof-of-concept metadevice with four-port wavefront manipulations is experimentally demonstrated, exhibiting distinct functionalities including spin- and frequency-dependent focusing, quad-beam radiation, anomalous reflections, and Bessel beam generation. Our finding of full-space spin-decoupled metasurfaces would be important for high-capacity communications, multifunctional radar detections, and other applications.

## 1 Introduction

Metasurfaces, composed of an assembly of deeply sub-wavelength artificial structures for complete control of light–matter interactions, have facilitated many novel electromagnetic (EM) functional devices [1, 2]. Compared with three-dimensional (3D) bulky metamaterials, metasurfaces possess many unique advantages such as lower insertion losses, easy fabrication, and negligible electrical thickness, while sustaining various exotic phenomena and applications such as anomalous refraction and generalized Snell’s laws [3], beam steering [4–6], diffusive scattering [7–10], ultra-thin cloak [11–15], orbital angular momentum generation [16, 17], and many others [18–20].

Particularly, metasurfaces are important for compact wireless and optical communications, which allow multitasking or multiple functionalities and significantly boosted information capacity [21, 22]. For example, anisotropic metasurfaces with polarization-sensitive EM responses can implement polarization multiplexing based on two orthogonal states, such as linear polarization (LP) basis or circular polarization (CP) basis, for promising satellite communication, especially for microwave frequencies [23]. Here, CP states (also known as spins of EM waves) have been commonly employed to integrate distinct functionalities into single and flat devices based on Pancharatnam–Berry (PB) phase [16, 24–28], which however exhibits intrinsic spin-locked phase profiles. To overcome this issue for more degree of freedom, decoupling two spins is realized by simultaneously employing geometric and propagation phases [18, 29], [30], [31], [32]. Nevertheless, the spin-decoupled strategy only operates in half space (i.e. either transmission or reflection region), showing limited capacity. Very recently, full-space wave control of a CP with a multilayer structure has been demonstrated [33–37], which unfortunately exhibits a spin-locked phase. To date, full-space spin-decoupled EM manipulation remains elusive and is rarely demonstrated.

**Figure 1:**
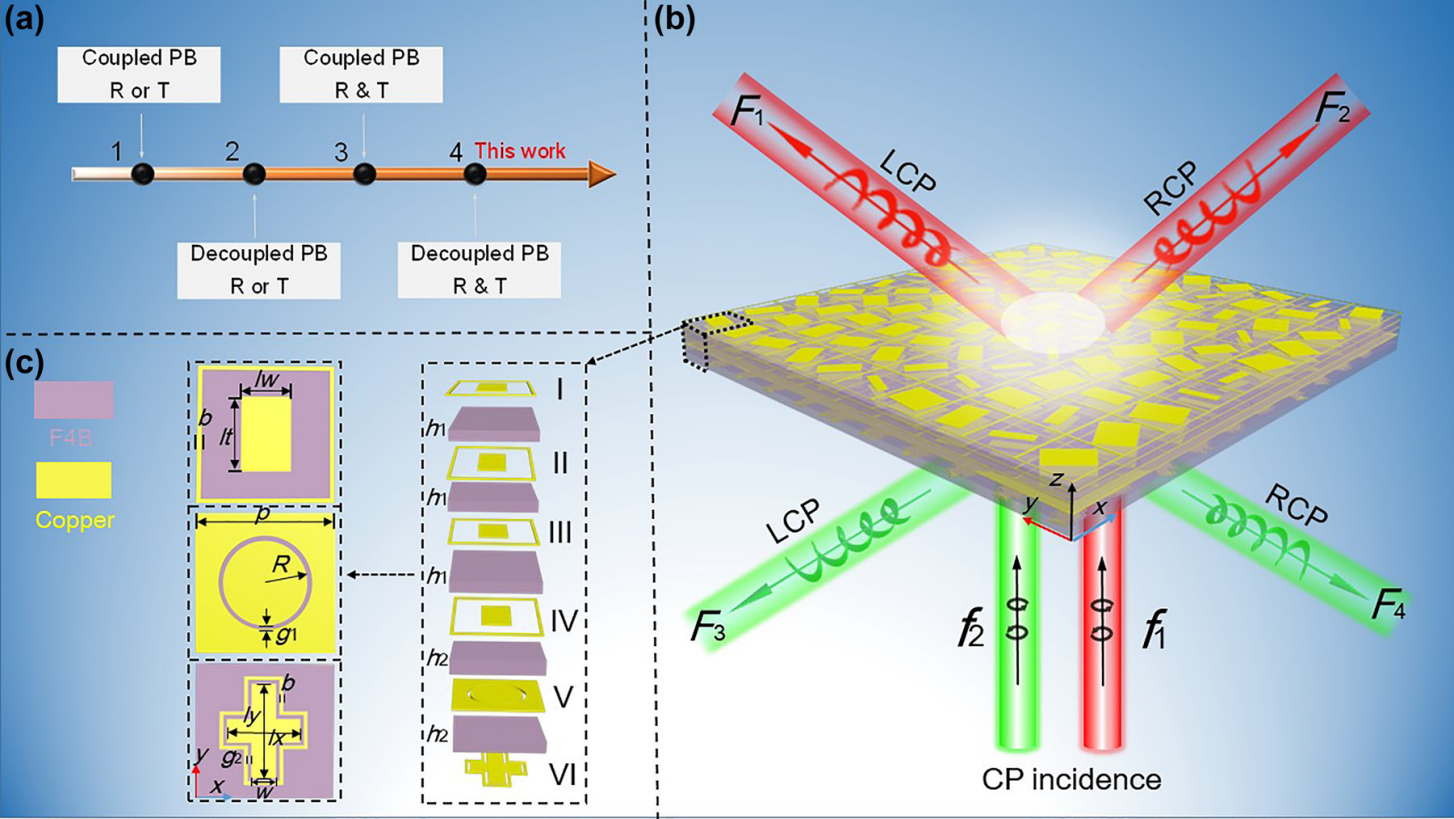
Illustration of (a) evolution and (b) advanced functions of our full-space spin-decoupled multichannel metasurface by using (c) proposed mode-decoupled meta-atom. The function *F*_1_ is achieved at frequency *f*_1_ under LCP wave excitation and (light red), denoted as the eigenchannel (*σ*_−_; *f*_1_). Similarly, *F*_2_, *F*_3_, and *F*_4_ exhibit the information channels (*σ*_+_; *f*_1_), (*σ*_−_; *f*_2_), and (*σ*_+_; *f*_2_). The pixel period is *p* = 12 mm. The widely available F4B dielectric board with *ε*_r_ = 2.65 and a loss tangent of 0.001 was used as the five-layer substrate. The thicknesses of the upper four-layer and bottom substrates were *h*_1_ = 1.5 mm and *h*_2_ = 2 mm, respectively. The other geometric parameters are *b* = 0.2, *R* = 4, *g*_1_ = 0.3, *g*_2_ = 0.2 and *w* = 2.4 mm. The length of crossbar/quadrangular patch structures along *x* and *y* directions is *l*_*x*_/*l*_*w*_ and *l*_*y*_/*l*_*t*_, respectively.

Here, we show a spin-decoupled multitasking strategy with high-efficiency CP wave control in full space. Distinct from any previous literature including unlocked dual-spin [38] and chirality-selective [39–42] metasurfaces, here we exploit an asymmetric meta-atom with non-interleaved cascading along vertical layered direction. Such a metasurface supports dual sets of spin-decoupled channels across two wavelengths in transmission and reflection mode (Figure 1(a)), respectively, and thus manifests particularly different functionalities as we experimentally demonstrated. Our distinct strategy promises many fascinating applications in fifth generation (5G) communications and paves a way for high-efficiency devices with unprecedented data capacity.

## 2 Results and discussion

### 2.1 Theoretical concept and meta-atom design

As shown in Figure 1(b), our proposed metasurface achieves quad-port wavefront control for CP wave incidence at two well-separated frequencies and in transmission and reflection mode, respectively. The quad-port versatile independent functionalities (*F*_1_, *F*_2_, *F*_3_, and *F*_4_) require arbitrary different phase profiles (*φ*_1_, *φ*_2_, *φ*_3_, and *φ*_4_) and hence unlocked dual-spin multiplexing and crosstalk-free dual-frequency multiplexing are necessary. To illustrate working principle, we take transmission mode analyses as an example. Under *x*- and *y*-polarized wave excitation, the EM response of a meta-atom with mirror symmetry along *x*- and *y*-direction can be described through transmission/reflection matrix *T*/*R* [32].

(1) $$\left(\begin{array}{c} E_{x}^{\text{out}}\\ E_{y}^{\text{out}} \end{array}\right)=T\left(\begin{array}{c} E_{x}^{\text{in}}\\ E_{y}^{\text{in}} \end{array}\right)=R\left(-\psi \right)\left(\left.\begin{array}{c} e^{\text{i}\varphi_{x}}\;\;\;\;\;\;\;0\\ 0\;\;\;\;\;\;\;\;\;e^{i\varphi_{y}} \end{array}\right)\right.\;R\left(\psi \right)\left(\begin{array}{c} E_{x}^{\text{in}}\\ E_{y}^{\text{in}} \end{array}\right)$$

Here, $E_{x/y}^{\text{in}}$ / $E_{x/y}^{\text{out}}$ denotes input/output electric field polarized along *x*-/*y*-direction, *φ*_*x*_/*φ*_*y*_ is transmission phase, *R* is rotation matrix related to rotation angle of *ψ*, while *T* = $\left(\begin{array}{c} t_{xx}\;\;\;t_{yx}\\ t_{xy}\;\;\;t_{yy} \end{array}\right)$ . For spin-independent phase control, the transmission matrix of the metasurface should satisfy.

(2a) $$T\left|k^{+}\right.\rangle ={\text{e}}^{\text{i}\varphi^{+}}\left|k^{+}\right.\rangle^{*}$$

(2b) $$T\left|k^{-}\right.\rangle ={\text{e}}^{\text{i}\varphi^{-}}\left|k^{-}\right.\rangle^{*}$$

where $\left|k^{+}\right\rangle =\left[\begin{array}{c} \kappa_{1}^{+}\\ \kappa_{2}^{+} \end{array}\right]$ and $\left|k^{-}\right\rangle =\left[\begin{array}{c} \kappa_{1}^{-}\\ \kappa_{2}^{-} \end{array}\right]$ represent two arbitrary orthogonal polarization states, *φ*_+_ and *φ*_−_ are corresponding required phase, * is complex conjugate, and + and − denote RCP and LCP wave, respectively. For CP wave, by combining equations (1) and (2), the relationship between required dynamic and geometric phases *φ*_*x*_, *φ*_*y*_, and *ψ* and functional phases is obtained as follows (see derive process detailed in Ref. [43]):

(3a) $$\psi =\left(\varphi_{+}-\varphi_{-}\right)/4$$

(3b) $$\varphi_{x}=\left(\varphi_{+}+\varphi_{-}\right)/2$$

(3c) $$\varphi_{y}=\left(\varphi_{+}+\varphi_{-}\right)/2+\pi$$

From this equation, we conclude that spin-independent phase modulation can be achieved by tuning transmission phase properties (*φ*_*x*_ and *φ*_*y*_) and rotation angle (*ψ*) of the meta-atom. Similarly, above criteria is also suitable for reflection mode. Therefore, it is crucial for us to implement spin-decoupled kaleidoscopic wavefront control in both transmissive and reflective modes. In the following, we will implement this target by integrating these dual sets of spin-decoupling at two frequencies.

To minimize spin and mode crosstalk among four information channels, a meta-atom should attain high reflectivity and transmittance with full 2π phases at its operation frequencies. Hence, we propose a high-efficiency spin-decoupled meta-atom for multitasking strategy in both *R* and *T* schemes, as shown in Figure 1(c). For practical design, we resort to four-layered square patch structures (layers I, II, III, and IV), one-layer frequency selective surface (FSS) (layer V), and one-layer crossbar (layer VI) in a composite meta-atom with negligible crosstalk at low and upper frequencies of *f*_1_ and *f*_2_ (see the inset of Figure 1(c)), where six metallic pattern layers are separated by five F4B substrates. Here, layer V is designed as a circular-slot FSS structure to separate transmission and reflection modes. In previous work, interleaved configurations typically suffer from issue of mode cross-talk and thus low operation efficiency. Therefore, a non-interleaved meta-atom is employed here to suppress spin and mode crosstalk, which is essential to achieve spin and frequency multiplexing [18]. Since a single metallic layer exhibits very low transmission rate and limited phase cover, the four-layer cascaded quadrangular patches with square rings (I, II, III, and IV) are exploited to form Fabry–Perot resonance and thus achieve a full 360° phase cover and high transmission rate. In addition, the square ring accompanied with metallic quadrangular patch generates dual operation modes and thus considerably extends phase coverage. Layer VI is designed as a crossbar structure with a closed loop to achieve high reflectivity and phase modulation with a 2*φ* cover.

**Figure 2:**
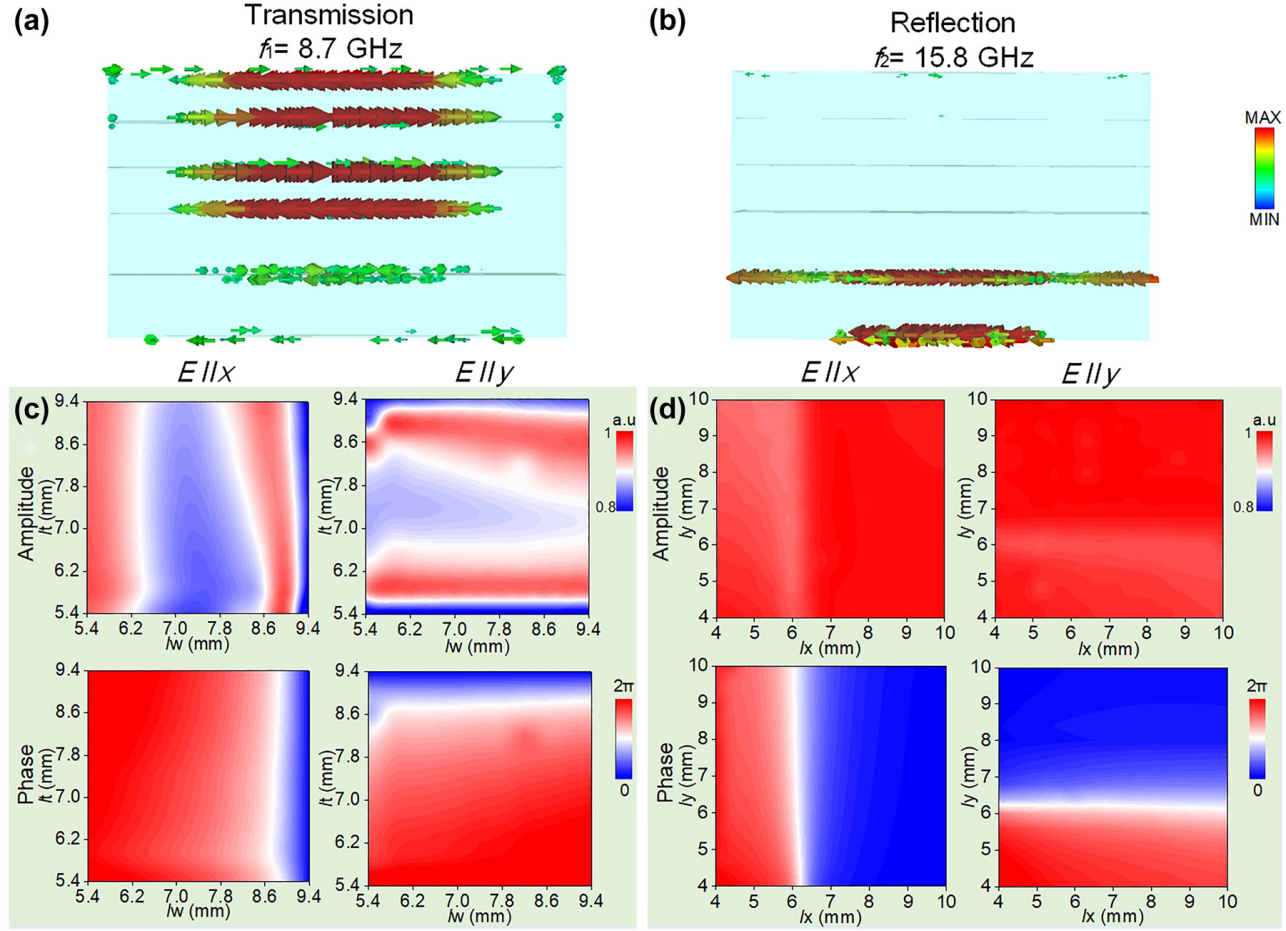
Current distribution and EM response of the meta-atom with different parameters at *f*_1_ and *f*_2_, respectively. Current distribution of the meta-atom for LCP incidence along *z* direction at (a) *f*_1_ and (b) *f*_2_. Amplitude and phase responses of co-polarization for *x* and *y* LP wave incidence as a change of different geometrical parameters (*l*_*w*_ and *l*_*t*_, *l*_*x*_ and *l*_*y*_) at *f*_1_ and *f*_2_ in (c) transmission and (d) reflection modes, respectively.

To further verify the physical origin of each mode, current distributions are also calculated. As illustrated in Figure 2(a), under LCP wave excitation, the current is mainly located on the quadrangular patch, accounts for a good transmission window at *f*_1_ = 8.7 GHz due to mutual interaction among four-layered patch. In sharp contrast, a strongly induced current is excited in Figure 2(b) at *f*_2_ = 15.8 GHz and generates a strong resonance between layer V and VI, indicating that the crossbar gives rise to the reflection mode.

We performed finite-difference-time-domain (FDTD) calculations to characterize our devices. The amplitude and phase mapping related to parameters (*l*_*w*_ and *l*_*t*_, varying from 5.4 to 9.4 mm) is shown in Figure 2(c), where the transmission amplitude maintains a high value above 0.8 at *f*_1_ = 8.7 GHz for *x*- and *y*-polarized incidence, respectively. Meanwhile, the transmission phase achieves full 2π coverage. Similarly, in Figure 2(d), the reflection amplitude and phase mapping are also obtained by changing *l*_*x*_ and *l*_*y*_ within 4–10 mm for *x*- and *y*-polarized waves, respectively, whereas reflection amplitude is near 1 while reflection phase covers a full 2π range at *f*_2_ = 15.8 GHz. In addition, favorable isolation between two modes is also observed by comparing calculated EM response in different cases, confirming the full-space independent wave manipulation with suppressed mode crosstalk; see more details in Supporting Information Figure S1. Therefore, we can easily get arbitrary phase modulation in both transmission and reflection modes for any desired functionalities in both spin channels and full space. In practice, the frequency ratio and periodicity of proposed meta-atom can be adjusted according to on-demand applications, which can be evidenced in Figure S2.

### 2.2 Full-space quad-channel multichannel metaplexer

As a proof-of-concept demonstration, we design and fabricate a quad-channel spin-multiplexed metasurface, termed as a metaplexer hereafter, by implementing focusing (*F*_1_) and quad-beam emissions (*F*_2_) under LCP and RCP states at transmission mode of *f*_1_ while manifesting beam deflection (*F*_3_) and Bessel beam (*F*_4_) at reflection mode of *f*_2_. Here, the designed metaplexer is composed of 27 × 27 position-varied meta-atoms and occupies a square area of *D* × *D* = 324 × 324 mm^2^. For experimental verification, we adopt standard printed circuit board technology to fabricate sample.

#### 2.2.1 Spin-decoupled wavefront controls in transmission mode

**Figure 3:**
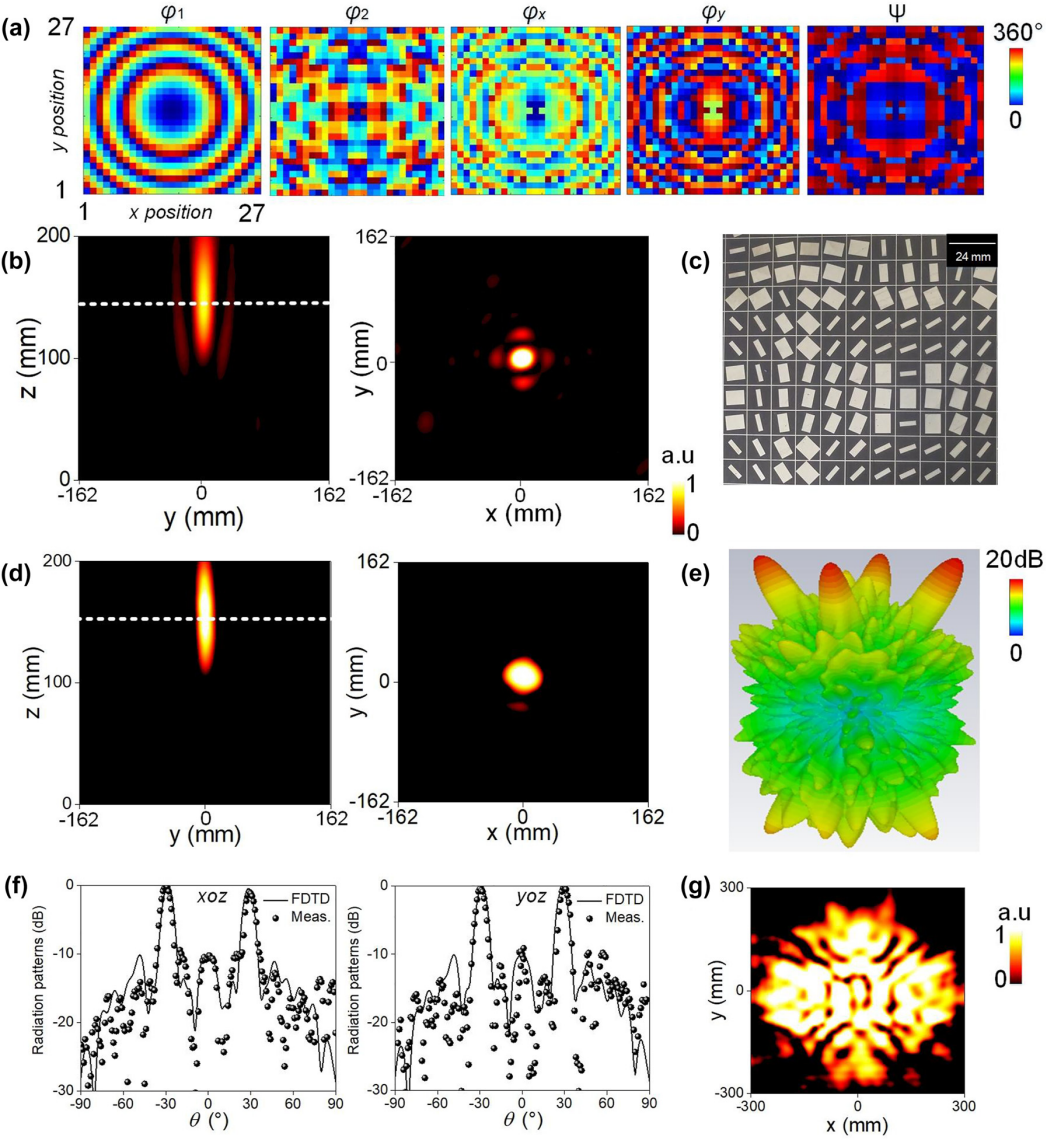
Characterization of the quad-information multitasked metaplexer with functions of focusing (*F*_1_) and quad-beam emissions (*F*_2_) in LCP and RCP channel of transmission mode at *f*_1_. (a) Synthetic phase for *F*_1_ and optimized phase for *F*_2_ based on alternating projection method, and the derived dynamic and geometric phases of *φ*_*x*_, *φ*_*y*_, and Ψ to decouple *F*_1_ and *F*_2_. (b) FDTD calculated near-field intensity on *y*–*z* (*x* = 0 mm) and *xoy* (*z* = 150 mm) plane to illustrate *F*_1_. (c) Zoom-in view of the fabricated sample. (d) Experimental near-field intensity on *y*–*z* plane (*x* = 0 mm) and *x*–*y* plane (*z* = 150 mm) by scanning areas of 324 × 200 and 324 × 324 mm^2^ in steps of 5 mm to reveal *F*_1_. (e) FDTD calculated 3D far-field radiation patterns for *F*_2_. (f) Comparison of FDTD calculated and experimental cross-section radiation patterns in *x*–*z* and *y*–*z* planes. (g) Experimental near-field intensities in *x*–*y* plane placed 110 mm away from the metaplexer at *f*_1_.

In Figure 3(a), we provide target phase profiles (*φ*_1_ and *φ*_2_) of *F*_1_ and *F*_2_, while *φ*_x_, *φ*_y_, and Ψ are derived by following above theoretical strategy. Here, the alternating projection method is utilized to synthesize phase pattern for *F*_2_ and the detailed process to obtain the appropriate geometrical parameters and rotation angles was presented in Ref. [32]. As a demonstration, we calculated near-field diffraction intensity on *y*–*z* and *x*–*y* planes with FDTD simulations. The results are shown in Figure 3(b), where a bright spot at the center of *x*–*y* plane with *z* = 150 mm (i.e. the focal length) indicates an excellent focusing performance of LCP channel at *f*_1_. This also agrees well with the experimental demonstration shown in Figure 3(c) and (d). According to the focusing efficiency defined by $\eta_{\text{foc}}=\frac{p_{\text{tra}}}{p_{\text{tot}}}\times \frac{p_{\text{foc}}}{p_{\text{tra}}}$ , the relative efficiency of the focusing lens is about 92 % for FDTD simulations and 89 % in measurement case. Here, *p*_tra_/*p*_tot_ represents the ratio between the power carried by the totally transmitted waves and that of the incident one, and *p*_foc_/*p*_tra_ is defined by the ratio between the power taken by the focal point and that of focal plane.

Multi-beam antenna arrays promise great potential in radar, satellite communication, and multi-input and multi-output systems. Here, we further realize quad-beam radiation under RCP wave excitation at *f*_1_. The detailed design method and numerical setup has been shown in Supporting Information Figure S3. The numerical far-field transmission pattern reveals that four pencil beam appears approximately at four designed directions (*θ*, *φ*) = (30°, 0°), (30°, 90°), (30°, 180°), and (30°, 270°), which confirms feasibility of the proposed strategy (Figure 3(e)). The quad-beam radiation property is also evidenced by the detailed cross-section radiation patterns in two principal planes described in Figure 3(f), where two main beams are deflected to *θ* = 30° and −30° and exhibit a reasonable agreement between FDTD simulations and experiments. Moreover, side-lobes remain a low level of below −10 dB, which guarantees high performance. Such an excellent quad-beam radiation is also supported by the measured near-field intensity distribution shown in Figure 3(g), where four spots are located in four different positions, conforming the characteristics of quad-beam radiation. The measured aperture efficiency is 33 % for the quad-beam emission, which coincides with 35 % in FDTD calculations. The averaged numerical transmission rate is 85 % (84 %) under LCP (RCP) wave excitation, respectively. All results demonstrate that the proposed metaplexer indeed achieves an independent phase transmission modulation under two orthogonal spin states at *f*_1_.

#### 2.2.2 Spin-decoupled wavefront controls in reflection mode

In the following, we demonstrate other functions, i.e., beam deflection (*F*_3_) and Bessel beam generation (*F*_4_) in reflection mode under LCP and RCP wave excitation, respectively at *f*_2_ by using our multichannel metaplexer.

**Figure 4:**
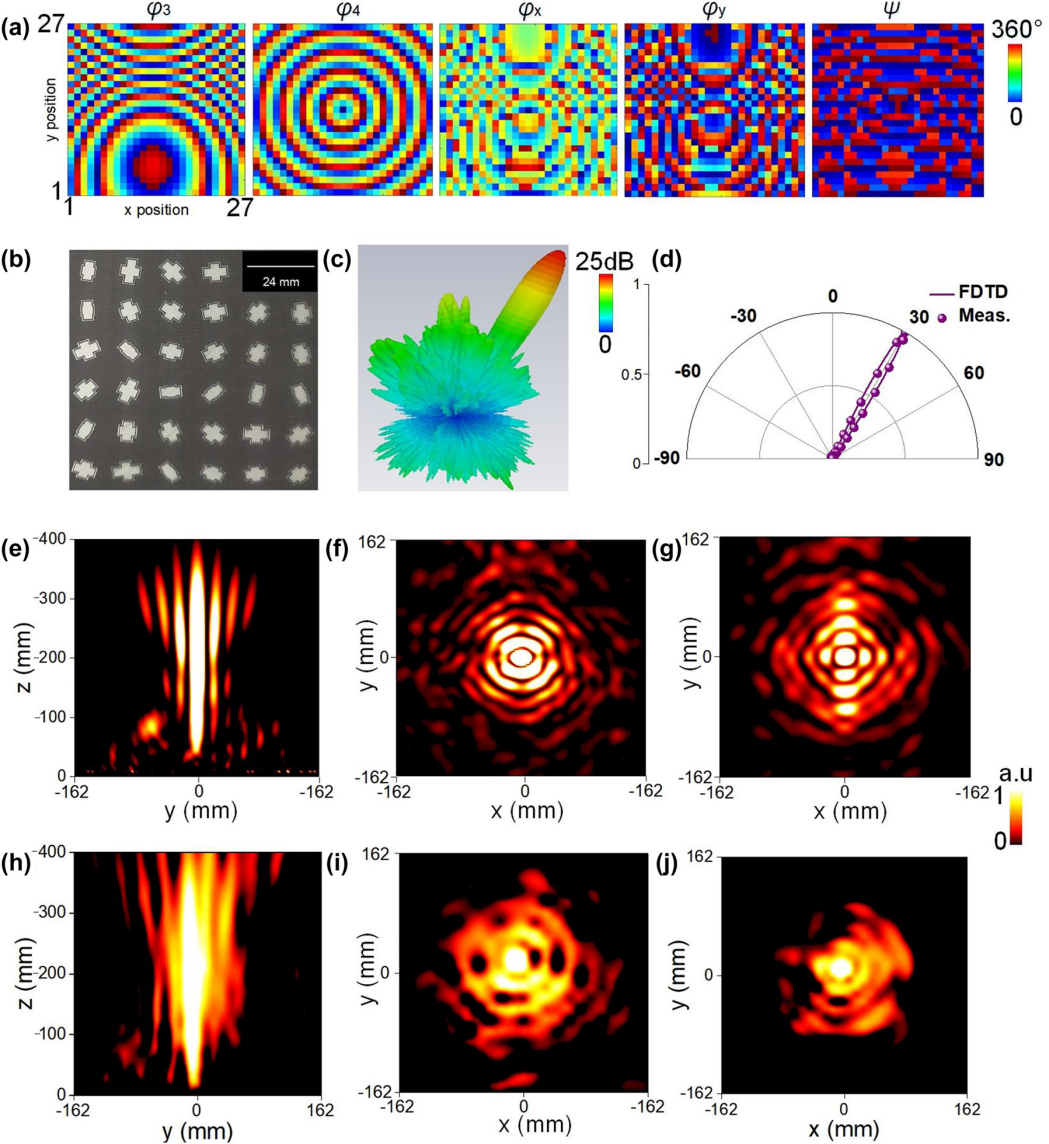
Characterization of the quad-information multitasked metaplexer with functions of beam deflection (*F*_3_) and Bessel beam (*F*_4_) in LCP and RCP channel of reflection mode at *f*_2_. (a) Synthetic phase patterns for *F*_3_ and *F*_4_, and the derived dynamic and geometric phases of *φ*_*x*_, *φ*_*y*_, and Ψ to decouple *F*_3_ and *F*_4_. (b) Zoom-in view of the fabricated sample. (c) FDTD calculated 3D far-field radiation pattern for *F*_3_. (d) Comparison of FDTD calculated and experimental cross-section radiation patterns in *x*–*z* plane. FDTD calculated near-field intensity on (e) *y*–*z* plane (*x* = 0 mm) and *x*–*y* plane of (f) *z* = 200 mm and (g) *z* = 300 mm to illustrate *F*_4_. Experimentally measured near-field intensity on (h) *y*–*z* plane (*x* = 0 mm) and *x*–*y* plane of (i) *z* = 200 mm and (j) *z* = 300 mm to illustrate *F*_4_.

The desired decoupled phase (*φ*_3_ and *φ*_4_) of *F*_3_ and *F*_4_ is shown in Figure 4(a), more details can be referred to Figure S4 in Supporting Information. In our design, the metaplexer is excited by a CP horn radiating LCP wave and the distance between it and the CP horn is set as *F* = 192 mm to suppress side-lobes, which guarantees highly directive beam generation. Here, the direction of beam is predefined in *θ* = 30°, *φ* = 0°. The fabricated sample and calculated 3D far-field patterns are shown in Figure 4(b) and (c), respectively. A pencil beam with extremely low side-lobes is precisely directed in predesigned direction. Numerically calculated and experimentally measured far-field scattering patterns at *φ* = 0° cross plane are plotted in Figure 4(d), showing a clear deflection beam with *θ* = 30°. Here, a slight deviation may be caused by the finite size effect, fabrication error, and background noise. The conversion efficiency of beam deflection is simulated and measured as 91.2 % and 90.1 %, respectively.

Bessel beam, as a classical non-diffraction beam, owns general advantages of long energy transmission distance and wide focusing range compared with other types, promising great potential in communication systems [44]. Here, we demonstrate a Bessel beam generation in RCP channel at *f*_2_. To generate a Bessel beam using metasurfaces, the abrupt phase profile should be in a cone shape. The phase distribution (*φ*_4_) described as: $\varphi =\frac{2\pi }{\lambda }\sqrt{{\left(mp\right)}^{2}+{\left(np\right)}^{2}}\sin\left(\beta \right)$ is shown in Figure 4(a), where *β* = 15° is base angle of equivalent axicon, *λ* is wavelength, *p* is the period of meta-atom and *m* (*n*) denotes the number of each meta-atom away from the center of metaplexer along *x*- (*y*-) axis. The calculated near-field intensity in *y*–*z* plane in Figure 4(e), shows a nondiffraction beam with excellent performance with a propagation length longer than 15*λ*. Furthermore, corresponding electric field intensity (of reflection) in *x*–*y* plane at *z* = 200 and 300 mm above the metaplexer is illustrated in Figure 4(f) and (g), respectively, where a bright central maximum appears in the center, exhibiting a good zero-order Bessel beam. Such a good performance is also supported by measurement results, as shown in Figure 4(h)–(j). In Figure 4(h), long-distance non-diffracting propagation behavior is observed, which coincides well with characteristics of a Bessel beam. In addition, as shown in Figure 4(i) and (j), a bright spot with maximum energy is clearly observed at the center of *x*–*y* planes of *z* = 200 and 300 mm, respectively, verifying a long energy propagation Bessel beam. Further simulation and experimental results at *z* = 100 and 400 mm are provided in Figure S5, all exhibiting similar patterns and confirming the non-diffraction long energy propagation property of zero-order Bessel beam. The averaged numerical reflection rate is about 95 % (96 %) under LCP (RCP) wave excitation, respectively. The efficiency of the Bessel beam is measured as 89 % while is calculated as about 91 % in FDTD simulations. All results clearly illustrate kaleidoscopic wavefront manipulation in four information channels based on spin-frequency multiplexing in full space, further verifying our concept and design. Moreover, we further explore the polarization purity of each designed functionality by investigating field distributions at its co-polarized spin state, see Figures S6 and S7.

## 3 Conclusions

We have proposed and demonstrated a full-space versatile wavefront manipulation based on spin-decoupled multichannel metasurface. In this regard, a composite meta-atom of six metal layers and five substrate layers was devised, in which the middle FSS structure is utilized to guarantee upper four-layers quadrangular patch with square rings working in transmission mode at *f*_1_ and bottom crossbar with a closed ring in reflection mode at *f*_2_, respectively. Thanks to elegant design for a suppressed spin and mode crosstalk, four information channels can be controlled independently in two orthogonal CP states, leading to independent full-space spin-decoupled kaleidoscopic wavefront manipulation. As proof of concept, a well-designed multitask metaplexer that exhibits four independent functionalities was experimentally demonstrated. Both numerical and experimental results qualify our proposed full-space strategy as a solid platform to realize spin- and mode-multiplexing systems with high information capacity, even our findings can be readily extended to more diverse wavefront modulations by introducing more eigenchannels associated with each piece of information, such as incidence direction and wavevector. We believe that our proposed full-space multitask metaplexer should provide new avenues and more degree of freedoms in designing multifunctional devices with high data capacity, promising great potential applications in the next-generation wireless communication systems.

## 4 Experimental characterization

**Figure 5:**
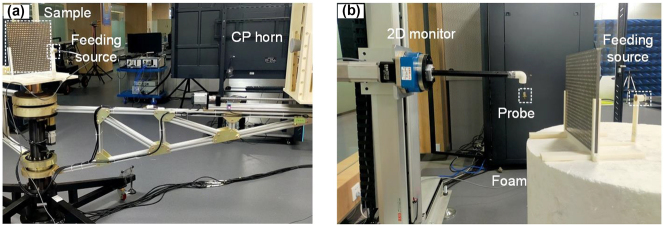
Experimental setups for (a) near-field and (b) far-field measurements.

To experimentally demonstrate our concept, a sample was fabricated by using the standard printed circuit board technique, where six-layer metallic patterns were printed individually on five dielectric boards, then assembled together through adhesives and finally reinforced through a hot press. As shown in Figure 5(a), in beam deflection and quad-beam radiation far-field measurements, the fabricated sample was placed in center of a rotation platform, where a small size LCP or RCP spiral horn working at 4–18 GHz was employed as feeding source to excite the fabricated metaplexer. The metaplexer was aligned with feeding horn by a 3D-printed frame with a distance of *F* = 192 mm. Meanwhile, an LCP or RCP horn was selected as receiving antenna at a reasonable distance of 3 m to record EM field information (amplitude and phase) by freely rotating 180° with a step of 1° around the sample. The feeding horn and receiving antennas were connected to two ports of an AV3672B vector network analyzer. In Figure 5(b), for near-field measurements of quad-beam radiation, the metaplexer was excited by an RCP horn from forward incidence, and a 6 mm-long monopole antenna, functioning as the receiver, was placed at 240 mm away from the metaplexer on another side. To obtain a near-field intensity distribution in *x*–*y* plane, a monopole antenna was connected with a 2D electronic step motor which can move automatically in a maximum area of 0.3 m × 0.3 m with a step resolution of 5 mm. In Bessel beam near-field measurements, the metaplexer was launched by an RCP lens antenna. Similarly, a monopole antenna was selected as the receiver to obtain reflection field information in area of 162 mm × 162 mm and 162 mm × 400 mm with a step resolution of 5 mm. By altering relative position of the metaplexer and 2D monitor, near-field distribution in *y*–*z* and *x*–*y* planes can be obtained. In all reflection near-field contour maps, incident signal in free space was deducted from total fields.

## Supporting Information

Discussion for crosstalk among different modes; discussion for frequency ratio and periodicity of proposed meta-atom; detailed design method and numerical setup for quad-beam radiation; reflection beam deflection phase calculation; additional information for Bessel beam; discussion for purity of each designed functionality by examing the field distribution at its co-polarized spin state.

## Supplementary Material

Supplementary Material Details
