# Supplementary material for: Full-space spin-decoupled versatile wavefront manipulations using non-interleaved metasurface
Source: Nanophotonics. 2023 Jun 19;12(15):3149–58. doi: 10.1515/nanoph-2023-0171 (PMC11501214; doi:10.1515/nanoph-2023-0171)
Supplement: Supplementary file 1 — Supplementary Material Details [file j_nanoph-2023-0171_suppl_001.pdf]

Supporting Information

Full-space spin-decoupled versatile wavefront manipulations using non-interleaved metasurface

Chaohui Wang[1], He-Xiu Xu*[1], Guangwei Hu[2], Yi Liu[2], Tong Liu[1], Kun Wang[1], Fan Zhang[1], Jian Xu[1], Shuo Xu[1], Zhichao Pang[1]

[1] *Air and Missile Defense College, Air Force Engineering University, Xi'an 710051, China*

[2] *School of Electrical & Electronic Engineering, Nanyang Technological University, 50 Nanyang Avenue, Singapore 639798*

*****Corresponding Author

E-mail: hxxuellen@gmail.com (He-Xiu Xu)

1. **Discussion for crosstalk among different modes**
2. **Discussion for frequency ratio and periodicity of proposed meta-atom**
3. **Detailed design method and numerical setup for quad-beam radiation**
4. **Reflection beam deflection phase calculation**
5. **Additional information for Bessel beam**
6. **Discussion for purity of each designed functionality by examing the field distributions at its co-polarized spin state**

1. Discussion for crosstalk among different modes

Crosstalk among modes can certainly interfere with or even deteriorate the performance in different channels. Therefore, a meta-atom with low crosstalk among modes is crucial to accomplish independent EM modulation in fully separate modes. We explore their mutual interference by separately rotating the quadrangular patch of layers I to IV and the crossbar of layer VI, respectively. According to the PB phase modulation principle, output phase change twice as much as rotation angle under CP wave excitation. Here, "-" and "+" denote LCP and RCP waves, respectively. We set rotation angle range as 0° ~ 180° with steps of 30° to control variables. For transmission mode, EM response is shown in **Figures S1(a)** to **(c)**. In **Figure S1(a)**, for the meta-atom with different rotated quadrangular patches, transmission amplitude only appears slight fluctuation at a working frequency of 8.7 GHz but remained more than 0.8, and reflection amplitudes remained stable at 15.8 GHz. Simultaneously, transmission phase provided in **Figure S1(b)** exhibits a linear change trend with rotation angle Ψ_1 and reflection phase illustrated in **Figure S1(c)** did not change. For reflection mode, transmission amplitude at 8.7 GHz remains almost unchanged, and reflection amplitude exceeds 0.98 at 15.8 GHz (**Figure S1(d)**). The reflection phase presented in **Figure S1(e)** still maintains a linear trend with rotation angle Ψ_2, and the transmission phase illustrated in **Figure S1(f)** did not change. In a word, above calculation results demonstrate that the designed full-space meta-atom not only achieves a high transmission and reflection amplitude but also exhibits excellent isolation characteristics between two modes, which provides a solid foundation for subsequent independent manipulation of EM waves in different channels.

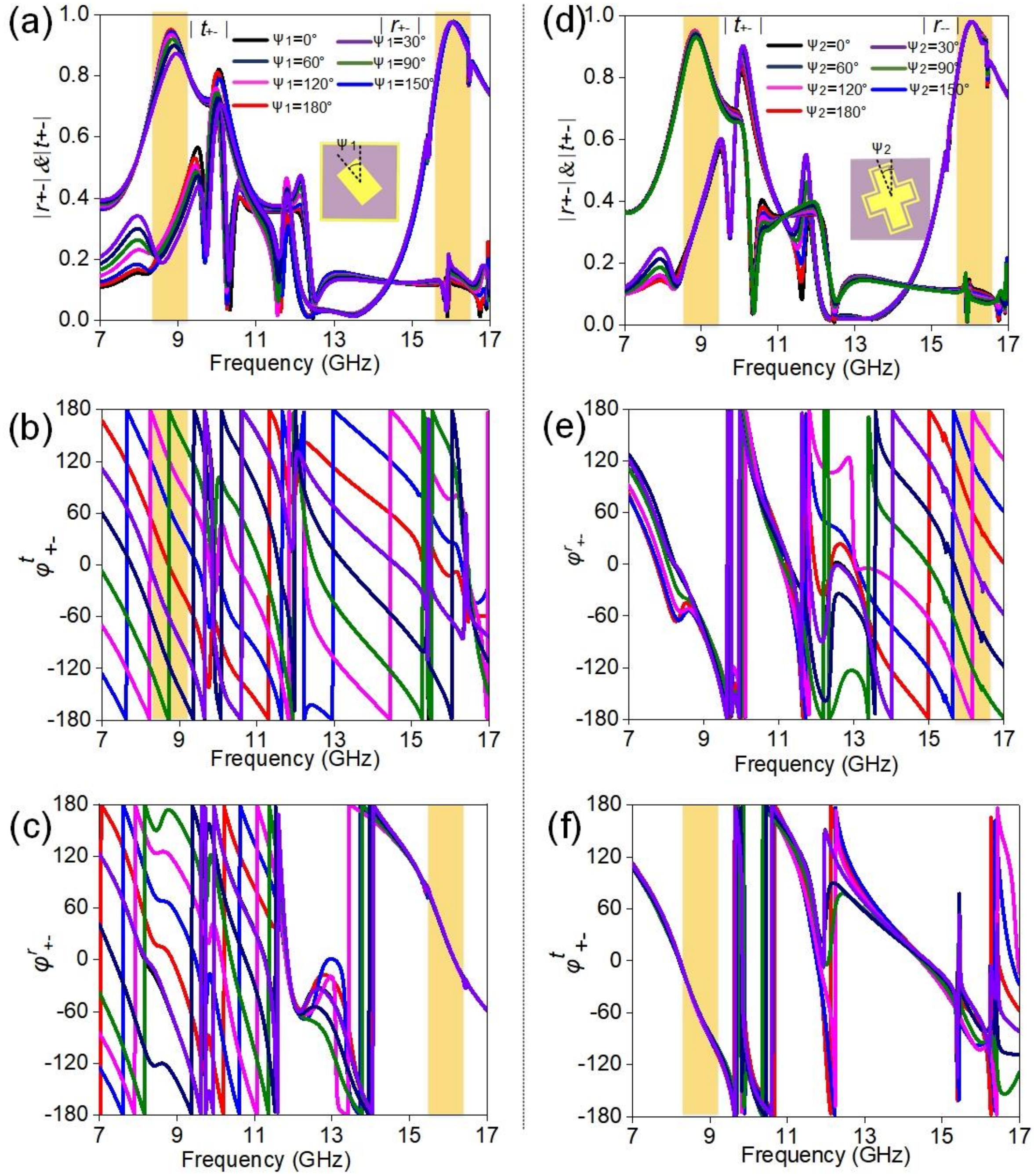

Figure S1 EM response of the meta-atom obtained by rotating individually: (a–c) four-layer quadrangular patch with square ring structures and (d–f) single-layer crossbar obtained when the counterparts were constant. We altered the rotation angle Ψ_1 (four-layer quadrangular patch structures) and Ψ_2 (single-layer crossbar) within the range of 0° ~ 180° with 30° rotation steps. (a) Reflection amplitude $|r_{--}|$ and transmission amplitude $|t_{+-}|$ at different Ψ_1. (b) Transmission phase φ^t_{+-} at different Ψ_1. (c) Reflection phase φ^r_{--} at different Ψ_1. (d) Reflection amplitude $|r_{--}|$ and transmission amplitude $|t_{+-}|$ at different Ψ_2. (e) Reflection phase φ^r_{--} at different Ψ_2. (f) Transmission phase φ^t_{+-} at different Ψ_2.

2. Discussion for frequency ratio and periodicity of proposed meta-atom

Generally, frequency ratio can be reduced by simultaneously increasing low frequency and reducing high frequency. For transmission mode, operation frequency mainly depends on the radius of a slot in ground and periodicity of meta-atom. Therefore, transmission window will move to more high frequency by reducing radius of the slot or periodicity. For reflection mode, working frequency can be shifted to a lower frequency by increasing thickness of substrate. Meanwhile, in practical applications, periodicity of the proposed meta-atom can also be appropriately reduced for antenna array designs by optimizing other geometrical parameters to guarantee excellent performance. To further demonstrate above, some numerical calculations are performed by adjusting the radius (R), thickness (h) of the substrate, and periodicity (p) of the meta-atom. The adjusted parameters are as follows: R = 3.5 mm, h = 2.5 mm, and p = 10 mm, respectively. From the simulated results shown in **Figure S2(a)** we can see that the transmission window is shifted from 8.7 GHz to 10.5 GHz and working frequency of reflection mode is decreased from 15.8 GHz to 12.5 GHz. In **Figures S2(b)** and **(c)**, phase exhibits a linear change trend with rotation angle in transmission and reflection mode. Frequency ratio is reduced from previous 1.8 to 1.19. Meanwhile, periodicity of proposed meta-atom is reduced from 0.64λ to 0.4λ. Therefore, frequency ratio and periodicity can be changed by optimizing geometrical parameters based on our strategy.

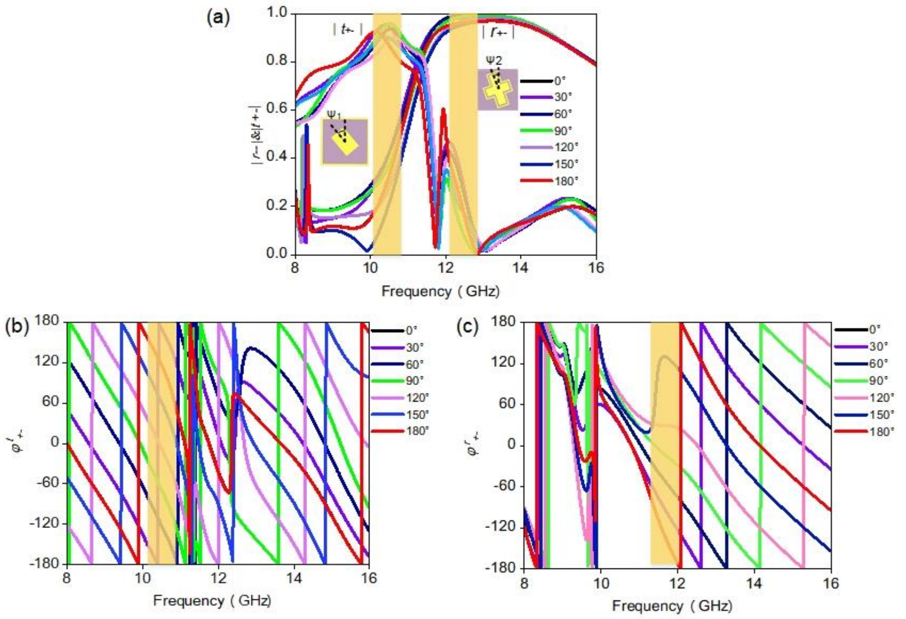

Figure S2 EM responses of the meta-atom by adjusting parameters of *R*, *h*, and *p*. (a) Transmission phase $\varphi^{t}{}_{+-}$ at different Ψ_1. (b) Reflection phase $\varphi^{r}{}_{--}$ at different Ψ_2.

3.Detailed design method and numerical setup for quad-beam radiation

For quad-beam radiation, we employ a CP feeding horn to excite the metasurface (see **Figure S3**). The feed horn is placed above the metasurface, and the distance between the feed horn and metasurface center is F=192 mm, which is beneficial to avoid much spillover from the source. In such a case, the alternating projection method is utilized to synthesize the phase pattern.

The main purpose of utilizing the alternating projection method to optimize the aperture phase distribution of quad-beam reflectarray is to search for the intersection between two sets, i.e. the set of possible far-field radiation patterns (set *A*) and the set of the target idealized patterns (set *B*) by employing a closed-loop iterative procedure. The possible far-field radiation patterns owe to the contributions ($\alpha_{m,n}$) of all meta-atoms at position (*m*, *n*) in reflectarray, which is determined by reflection amplitude and phase.

$$A \equiv \left\{ T : T(u,v) = \sum_{(m,n)\in I}^{N} \alpha_{m,n} e^{jk(p^{x}_{m,n}u + p^{y}_{m,n}v)} \right\} \tag{S1}$$

Here, *I* is the set of positions of all meta-atom, $u = \sin\theta\cos\varphi$ and $v = \sin\theta\sin\varphi$ are the angular

coordinates, $p^{x}_{m,n}$ and $p^{y}_{m,n}$ are positions of specific meta-atom along x and y direction, respectively.

To achieve presupposed quad-beam emissions and high-efficient work, the target idealized patterns need still to meet two mask requirements. In the first mark, we use the lower and upper bound values (M_L =0.707 and M_U =1) to respectively characterize the −3dB beam width and each main beam of target patterns, which is crucial to the multi-beam with uniform amplitude.

$$B \equiv \left\{ T : T(u,v) = M_L(u,v) \le \left| T(u,v) \right| \le M_U(u,v) \right\} \quad \text{(S2)}$$

In addition, we expect the side lobes to have a value as small as possible. Therefore, to suppress the side-lobe level, the radiated fields in the side-lobe region must meet the second mask requirements.

$$B \equiv \left\{ T : \left| T(u,v) \right| \le M_U \right\} \quad \text{(S3)}$$

A cost function to restrict the radiation level in the side-lobe area.

$$T_{adp} = \sum_{u^2+v^2 \le 1} \sum \left(\left| T(u,v) \right| - M_U \right)^2 \quad \text{(S4)}$$

Finally, as shown in the main text, excellent quad-beam radiation is achieved based on the optimized phase pattern.

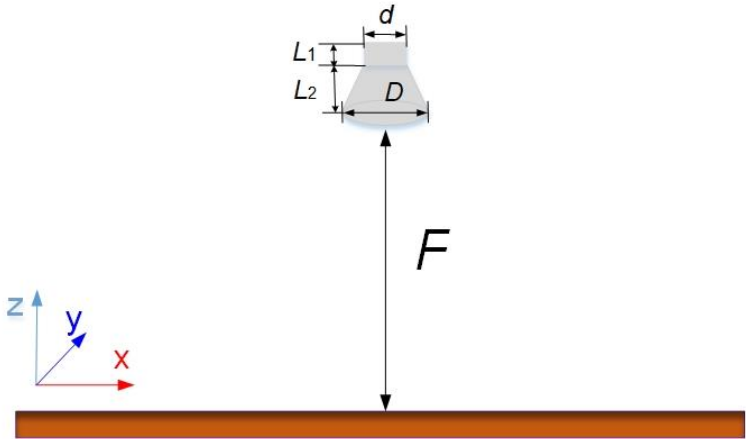

Figure S3 Numerical setup for the proposed metasurface with quad-beam radiation. The distance between the feeding horn and metasurface center is F = 192 mm. The physical parameters of the feeding horn are d = 17, D = 22, L_1 = 25, and L_2 = 30 (unit: mm).

4. Reflection beam deflection phase calculation

To achieve a high-gain directional beam with low side-lobes, we present a schematic diagram of the reflection beam deflection manipulation in **Figure S4**, where the plane wave is replaced by a feeding horn to excite the designed metasurface. According to the antenna array theory, if we expect a beam to be generated in a specified direction by the designed metasurface, then the local reflection phase of the meta-atom should satisfy the following equation:

$$\phi_{i,j}(\theta,\varphi) = \psi + k_0 r_{i,j} - k_0(x_{i,j}\sin\theta\cos\varphi + y_{i,j}\sin\theta\sin\varphi) \quad \text{(S5)}$$

where k_0 is the wavenumber in the free space at target frequency f_0, $r_{i,j}$ is the distance from the (*i*, *j*)th meta-atom to the feeding source, $\phi_{i,j}(\theta,\varphi)$ is the calculated reflection phase of the (*i*, *j*)th meta-atom, θ and φ are the elevation angle and the azimuth angle of the designed beam, respectively, $x_{i,j}$ and $y_{i,j}$ are the coordinates of the (*i*, *j*)th meta-atom, and ψ is an arbitrary initial phase. Here, the initial phase is set as 0°. In our design, the position of feeding source is fixed above the metasurface with a distance of *F* = 192 mm, which is beneficial for suppressing level of side-lobes and achieving a high gain beam.

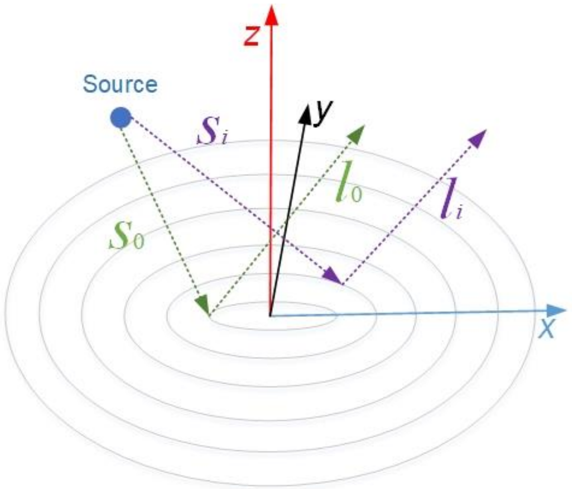

Figure S4 Schematic diagram of the proposed metasurface

5. Additional information for Bessel beam

Figure S5 gives the calculated and measured electric field intensity in *x*-*y* plane with *z* = 100 and 400 mm, respectively. As depicted in **Figure S5(a)**, energy is gathered in the center of the

plane, indicating a nondiffraction beam has been generated in the position of z = 100 mm. Such a physical phenomenon is also supported by the corresponding measured results shown in **Figure S5(c).** Similarly, in the plane of z = 400 mm, the energy convergence effect remains (see calculated results shown in **Figure S5(b)** and measured ones in **Figure S5(d)**), verifying the energy is converged in the range of 100 ~ 400 mm (15λ) along z-axis, which coincides with the nondiffraction long-distance propagation property of Bessel beam.

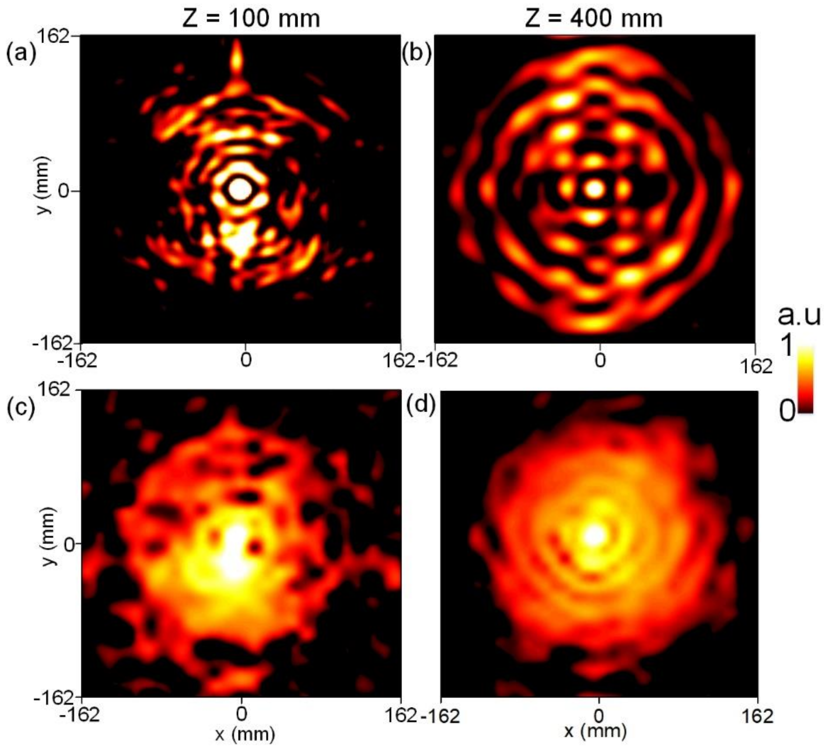

Figure S5 Additional information for the function of Bessel beam generation. FDTD calculated near-field intensity on x-y plane with (a) z = 100 mm and (b) z = 400 mm. Measured near-field intensity on x-y plane with (c) z = 100 mm and (d) z = 400 mm.

6. Discussion for purity of each designed functionality by examing the field distributions at its co-polarized spin state

Polarization purity determines working efficiency of devices, which is crucial to practical applications. Here, we explore polarization purity by calculating field distributions of the co-polarization states of each designed functionality. In **Figures S6**(a) and (b), for light focusing, near-field intensity exhibits disorder distribution and owns an extremely low level (near 0), which

demonstrates almost all transmission waves exist in the target spin channel. For quad-beam emission, we give FDTD calculated radiation patterns in the orthogonal spin states (**Figure S6**(c)), where both radiation patterns in *x*-*z* and *y*-*z* planes are below -10dB compared with ones in target spin channel, verifying a high polarization purity for the functionality of quad-beam emission. Similarly, as shown in **Figure S7**(a), the radiation patterns are also below -10dB, which is solid evidence to high polarization purity for the designed functionality F_3. In **Figures S7**(b) ~ (f), we give FDTD calculated near-field intensity of F_4 on *y*-*z* plane (x = 0 mm) and *x*-*y* plane of z = 100 mm, 200 mm, 300 mm, and 400 mm, respectively, where no phenomenon of Bessel beam was observed and near-field intensity exhibits a negligible level in the co-polarization channel, proving Bessel beam only exist in target polarization channel. In a word, all the above results demonstrate a high polarization purity of each designed functionality.

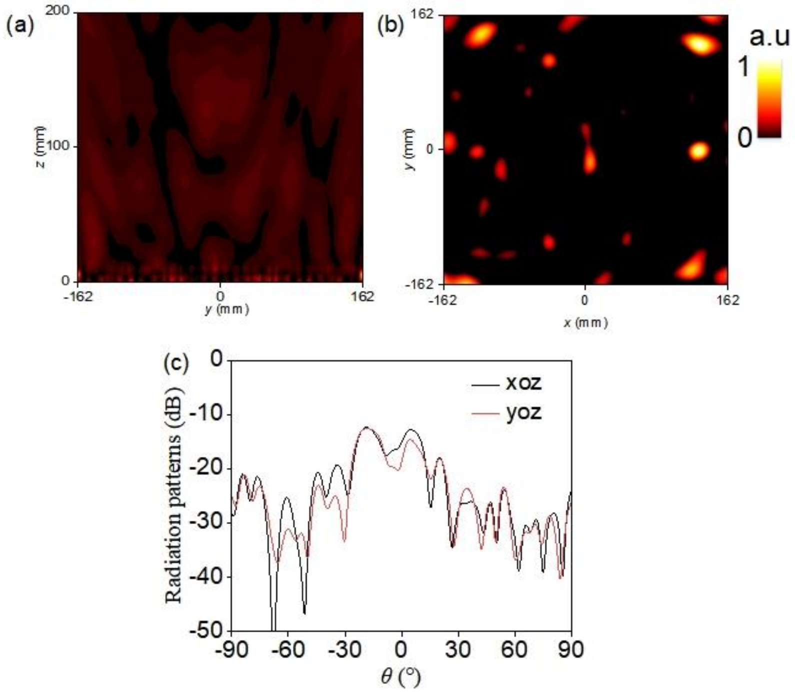

Figure S6 Characterization of the quad-information multitasked metaplexer with functions of light focusing (F_1) and quad-beam emissions (F_2) in the orthogonal spin channels. FDTD calculated near-field intensity of F_1 on (a) *yoz* (x = 0 mm) and (b) *xoy* (z = 150 mm). (c) FDTD calculated cross-section radiation patterns of F_2 in *xoz* and *yoz* planes.

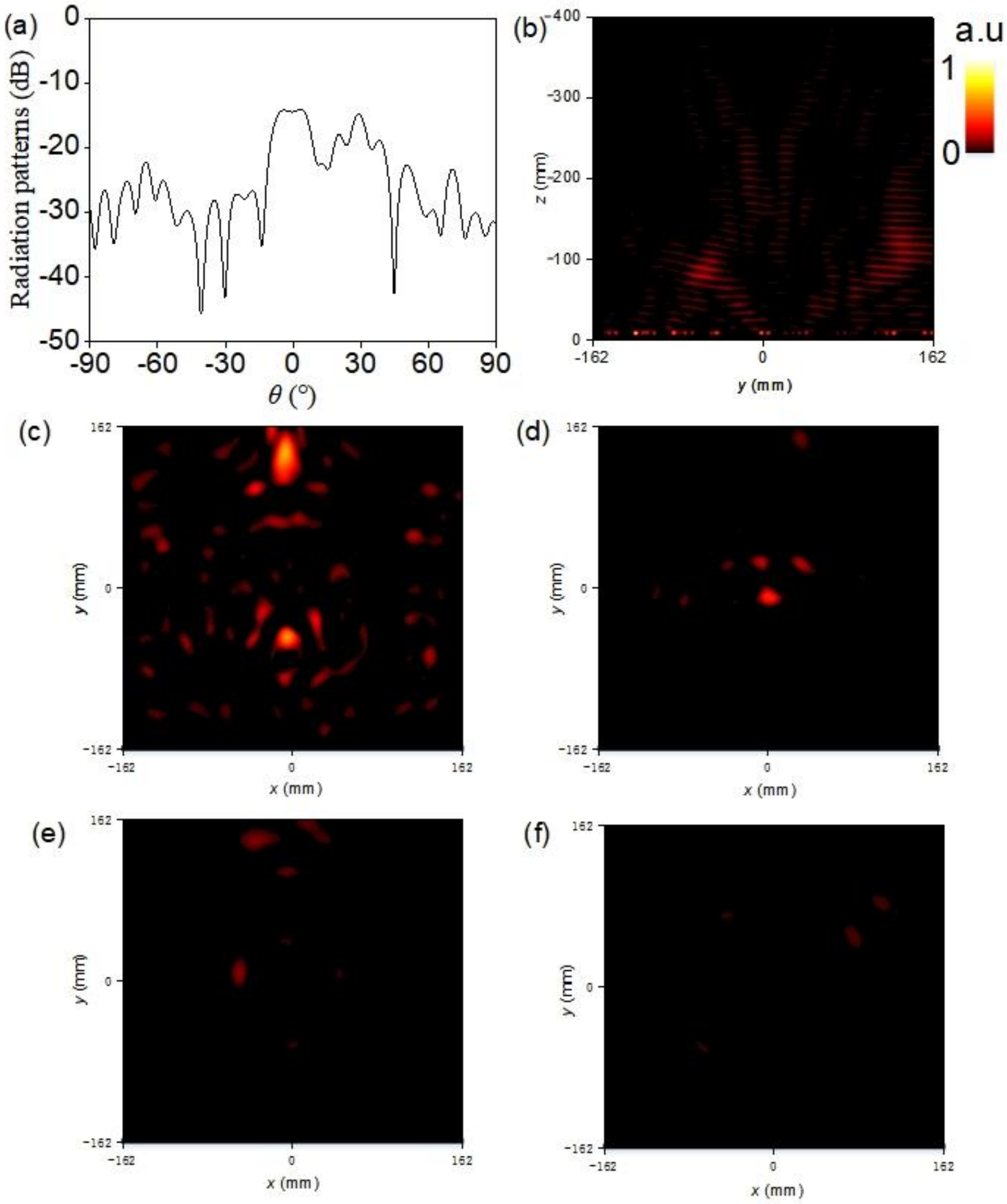

Figure S7 Characterization of the quad-information multitasked metaplexer with functions of beam deflection (F_3) and Bessel beam (F_4) in the orthogonal spin channels. (a) FDTD calculated cross-section radiation patterns of F_3 in *xoz* plane. FDTD calculated near-field intensity of F_4 on (b) *yoz* plane ($x = 0$ mm) and *xoy* plane of (c) $z = 100$ mm, (d) $z = 200$ mm, (e) $z = 300$ mm, and (f) $z = 400$ mm, respectively.
